# An effective universal protocol for the extraction of fructooligosaccharide from the different agricultural byproducts

**DOI:** 10.1016/j.mex.2023.102096

**Published:** 2023-02-26

**Authors:** Ramachandran Chelliah, Se Jin Park, Sungyoon Oh, Eunseok Lee, Eric Banan-Mwine Daliri, Fazle Elahi, Chae Rin Park, Ghazala Sultan, Inamul Hasan Madar, Deog Hwan Oh

**Affiliations:** a Department of Food Science and Biotechnology, College of Agriculture and Life Sciences, Kangwon National University, Chuncheon, Gangwon-do 24341, Republic of Korea; b Department of Food Science and Biotechnology, Kangwon Institute of Inclusive Technology (KIIT), Kangwon National University, Gangwon-do 24341, Republic of Korea; c Saveetha School of Engineering, (SIMATS), Chennai, Tamil Nadu 600124, India; d School of Natural Resources and Environmental Sciences, Kangwon National University, Chuncheon 24341, Republic of Korea; e Department of Computer Science, Aligarh Muslim University, Aligarh 202002, India; f Department of Pharmacology, Saveetha Dental College and Hospitals, Chennai, Tamil Nadu 600077, India; g Department of Biological Models, Institute of Biochemistry, Life Science Center, Vilnius University, Saul..tekio al. 7, 10257 Vilnius. Lithuania

**Keywords:** Oligosaccharide, Alkaline-based enzymatic extraction, High-performance liquid chromatography, Pre-column derivatization, Acetylation of extract, NMR, COSY, FTIR, PSA, SEM

## Abstract

Alternative bio-refinery technologies are required to promote the commercial utilization of plant biomass components. The fructooligosaccharide (FOS) obtained after hydrolysis of the hemicellulose fractions was mainly applied in the pharmaceutical and food industries. Agricultural bi-product is a rich constituent in dietary fibres, which have prebiotic effects on the intestinal microbiota and the host. Herein we explored the impact of FOS on microbiota modulation and the gut homeostasis effect. High fructooligosaccharide recovery was obtained using alkaline extraction techniques. The enzymatic method produced fructooligosaccharides with minor contamination from fructan and glucan components, although it had a low yield. But combining the alkaline and enzymatic process provides a higher yield ratio and purity of fructooligosaccharides. The structure of the fructooligosaccharide was confirmed, according to FTIR, ^13^C NMR, ^1^H NMR and 2D-NMR data. Our results could be applied to the development of efficient extraction of valuable products from agricultural materials using enzyme-mediated methods, which were found to be a cost-effective way to boost bio-refining value. Fructooligosaccharides with varying yields, purity, and structure can be obtained.

## Graphical abstract

**Figure:**
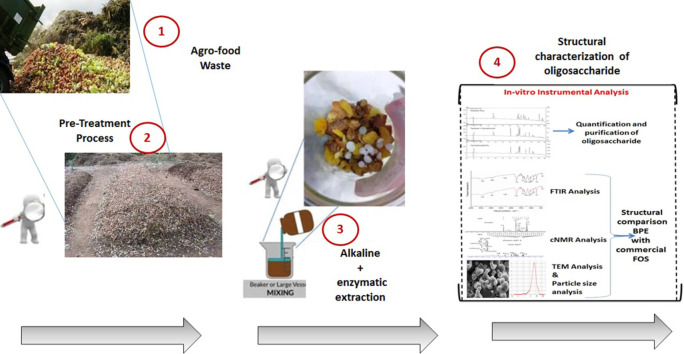

Specifications table

**Table:** 

**Subject Area**:	Agricultural and Biological Sciences
**More specific subject area**:	Bio-resource Technology
**Protocol name**:	Alkaline combined enzymatic method
**Reagents/tools**:	The banana peel was kept in a hot-air oven at 105 °C for 24 h, and after that, the dried banana peel was weighed to determine the moisture content.°C for 24 h. After drying, the peel was crushed into a fine powder, sieved through a 525 µm sieve, and based on winnowing, the husk was collected in a sterile container and stored at -20 ± 2°C. Chemical reagents that were used in the experiment primarily included analytical grade glacial acetic acid, 99% ethanol, hydrochloric acid (HCL), trifluoroacetic acid (TFA), hexane, deuterium oxide (D2O), glucoamylase (7000U) (Sigma, 9032-08-0), α-amylase (1000U) (Sigma 10102814001), and standard kestose, glucose; fructose; rhamnose, maltose, arabinose, inulin, sucrose, cellobiose, 2% glutaraldehyde and 2% paraformaldehyde, uranyl acetate and lead citrate.
**Experimental design**:	The methodology for extracting fructo-oligosaccharide from agricultural byproducts (banana peel) is briefly described in the paper, which also analyzes the structural characterization and physicochemical properties of oligosaccharides. Further, the protocol can enhance the yield ratio and wide application. The advantages of this technique are presented below: *Combined alkaline and enzymatically based extraction protocol. *Efficient validation of fructooligosaccharide (Kestose) based on structural characterization using instrumental analysis. **1. Preparation of byproduct samples** Banana (*Musa paradisiaca Linn*.) fruit was procured from a supermarket in Chuncheon, Gangwon-do, South Korea. Preparations of dry powdered banana peel were performed as described earlier [1]. **2. Sample preparation** **2.1. Preparation of total oligosaccharide extract from banana peel** Fresh bananas were purchased from Lotte Mart in Chuncheon, peeled, dried in the shade, and dried at 30 °C using a dry oven. The dried banana peel was homogenized into a fine powder using an experimental blender (Model Z272221) Waring Laboratory, Seoul, South Korea), 50 g of banana peel powder was defatted using the soxhlet apparatus (SciLab, South Korea) using hexane solvent at 70 °C [1]. The defatted sample was subjected to 100 mL sodium acetate buffer (0.2 M, pH 7.0 ± 2) and incubated with 1 mg of α-amylase (1000U) (Sigma 10102814001) at 95 °C for one hour. After cooling, the samples were subjected to 100 mg of glucoamylase (7000U) (Sigma, 9032-08-0) and heated at 55 °C for 48 h. The enzyme-treated sample was centrifuged (Union 32 Rplus, Incheon, South Korea) at 4000g for 20 min. Further de-starched samples were extracted using 1000 mL of distilled water (DW) at 25 °C for 2 h. The oligosaccharide precipitate was obtained using three volumes of 99% ethanol. Further, the crude oligosaccharide was lyophilized using a lyophilizer (IBK1400 RFD, Daejeon, South Korea) and stored at 4 °C until further use [1] (Fig. 1). **3. Isolation and identification of FOS (kestose) from BPOE** **3.1. Analysis of BPOE by High-performance liquid chromatography system coupled with Refractive Index Detector (HPLC-RID)** An HPLC system (Agilent 1260) ((Agilent Technologies, Palo Alto, CA, USA) was applied. Banana peel oligosaccharide extract (BPOE) and chicory derived standard kestose (GF2) are compared and analyzed [2]. The BPOE extract was analyzed using HPLC-RID to confirm the fructooligosaccharide (GF2, F8052 Sigma-Aldrich) derived from chicory. Carbohydrate content analysis was equipped with a C18 column (300 × 3.9 mm; Waters, USA), autosampler, online degassing device, double pump, column temperature controller, 1260 Infinity II Refractive Index Detector, and analytical workstation (Agilent Technology, Germany) to confirm the components of GF2 and BPOE derived from chicory. **4. Separation of kestose (GF2) using high-speed counter-current chromatography (HSCCC)** **4.1. Pre-column derivatization** The pre-column derivatization of *kestose (GF2)* was performed and the methodology was described by the following method described in Ref. [3]. Anhydridus Acetic (19 g, 0.4 mol) was added to a solution of BPOE (5 g) in pyridine (50 mL) at room temperature. After the reaction mixture was stirred overnight, the solution was concentrated under reduced pressure to obtain the raw product as yellow syrup, used for further purification [3]. **4.2. High-speed countercurrent chromatography (HSCCC) separation device** High-speed countercurrent chromatography (Tauto Biotech TBE-300A, Shanghai, China), which consists of a 150 m long multilayer coil with an inner diameter of 1.6 mm and a total volume of 300 mL, was employed to separate kestose (GF2) from the total oligosaccharide extract of banana peel. The β value of this preparative column ranged from 0.8 on the outside to 0.5 on the inside β = r / R, (where r is the turning radius or the distance from the coil to the holder shaft, R (*R* = 8 cm)). A Model NS-1007 constant-flow pump was applied to transfer the solvent into the column. A 30 mL loop manual sample injection was used to inject the sample into the column. The spectrum was measured at a wavelength of 250 nm, and the hydrogen ion concentration (pH) was confirmed using a pH meter (Hanna precision model p1242-Merck, Seoul, Korea). Chromatographic values ​​were obtained using a portable recorder and documented [4,5]. **4.3. Quantification of Partition Coefficient (ratio of the equilibrium concentrations of a dissolved substance in a two-phase system)** The KD value was determined by Ito [6]. The acetylated extract (ABPOE) (1 g) and GF2 derived from chicory were separately dissolved in 20 mL of a test tube, and 5 mL of each was completely flat, and a two-step solvent system was added. 1 mL of each of the upper and lower portions was evaporated through a nitrogen flow. The residue was diluted with methyl alcohol (CH3OH) (1 mL of 99.9%) to regulate the specific compound's KD value. KD = PA/AL (PA: peak area of ​​upper phase/ LP: lower phase) [7]. **4.4. Preparation of Sample solution and two-step solvent analysis** To obtain the sample solution, the Acetylated extract was liquefied in the mixed two-phase solvent system (LP: lower phase/ PA: peak area of upper, 1:1, v/v). Each solvent was transferred into the reparatory funnel to obtain the two-phase solvent system. The two phases were classified as follows, the upper phase (as the stationary phase) and the lower phase (as the mobile phase) [6].
	**4.5. Separation procedure** The separation procedures were described by Aruda et al. [8]. This was done using the method. The rotational speed of the apparatus was moved to a multilayer coil column at 800 rpm, 2 mL/min until the stationary phase (upper phase) was filled with a flow rate of 15 mL/min, and the mobile phase (lower phase) was moved at a flow rate of 2 mL/min. After reaching hydrodynamic equilibrium, the column product was continuously monitored using an Ultraviolet (UV) detector at 250 nm and fractionated at 5 min intervals using different fractions with 10 mL of both phases) was injected into the column through the injection valve. The residual solvent of the column was pushed out, and the residual ratio of the upper face was measured. The specific fractions were further analyzed using HPLC-ELSD, and the high-purity kestose (GF2) fraction was collected and vacuum dried. **4.6. Reduction reaction** The Purified acetylated kestose (GF2) was reduced based on [9]. The target substance was dissolved in sodium methoxide and methyl alcohol (3 g, 0.055 mol), then homogenized for two hours at 25 °C. The neutralized reaction was filtered using Amberchrom 50WX8 hydrogen form (H + form) and 200–400 mesh (Sigma – 217514, South Korea). The viscus solution was obtained and concentrated based on reduced pressure. Purification was performed using Sephadex LH 20 - Cross-Linked Dextran column. **5. Confirmation of kestose (GF2) using high-performance liquid chromatography-vaporization light scattering detector (HPLC-ELSD)** The fractions separated and purified fructooligosaccharides using HSCCC were finally analyzed using HPLC-ELSD [21,22]. The agilent system was applied for HPLC equipment (Agilent 1260 TCC, Seoul, South Korea), Agilent 1260 quat pump, Agilent 385-ELSD (Evaporative Light Scattering Detector), and Agilent workstations (Agilent, Palo Alto, CA, USA). Analysis conditions were as follows: Acetonitrile: water (75:25 v/v) as the mobile phase and separated at a 1 mL/min flow rate at 25 °C using a Xamide 100A column (250 × 4.6 mm, id). In addition, the concentration was expressed in mg/g. **6. Structural analysis and characterization of purified kestose (GF2)** **6.1. Carbon nuclear magnetic resonance (¹³C-NMR)** To discover kestose (GF2), a fructooligosaccharide target chemical that was isolated and purified from banana peel oligosaccharide extract, the following experiment was carried out (BPOE). In organic solvents, D_2_O and tetramethylsilane (TMS) were used as standard solvents for correcting the chemical shift using a 600 MHz NMR spectrometer (Bruker Avance I I-700, Germany). The standard product was chicory-derived fructooligosaccharide (GF2, F8052-Sigma-Aldrich, South Korea) [10]. **6.2. Proton nuclear magnetic resonance (^1^H-NMR)** The following experiment was performed to identify kestose (GF2). D_2_O was used as a standard solvent for correcting the chemical shift of 1H-NMR using a 600 MHz NMR spectrometer (Bruker Avance I I-700, Germany). Samples were analyzed for 3.5 s with a pulse of 90° at 30 °C. As a control, a chicory-derived standard kestose (GF2, F8052-Sigma-Aldrich, South Korea) was used, and resonance was measured by referring to the biological magnetic resonance data bank (https://bmrb.io/metabolomics/mol_summary/show_data.php?id=bmse001112) **6.3. Two dimensional (2D)-Homonuclear (H-H) J-resolved correlation spectroscopy (COSY)** COSY analysis was conducted through a 2D spectrum to confirm hydrogen and hydrogen bonding more reliably. It was run for 1 h, and the existing spectrum experiment used 2000 complex data points, and experiments were acquired and recorded for every 16 scans. In contrast, in the 2D spectrum experiment, 1000 complex data points and 200 scans each were recorded. The investigation was calculated by increasing and decreasing the length of each continuous pulse in a Gaussian method. The total pulse length was used the same as the method used in the previous experiment (66 ms) and proceeded concerning the previous research records [11]. **7. Morphological Characterization** **7.1. Particle size analysis (PSA**) The particle size of purified kestose (GF2) from BPOE was characterized by a particle size analyzer (SZ 100, Horiba, Japan). It was measured using powder microparticles, and the colloidal particle size in a dispersed state was measured. The Z-average diameter determined particle size data [12]. **7.2. Scanning electron microscopy (SEM)** The observation was made using a transmission electron microscope to compare the morphological properties of purified kestose (GF2) isolated from BPOE and standard kestose (GF2). First, the sample was fixed for two hours at room temperature in 0.1 M cacodylate buffer containing 2% glutaraldehyde and 2% paraformaldehyde containing 2% glutaraldehyde and 2% paraformaldehyde containing 2% glutaraldehyde and 2% paraformaldehyde containing 2% glutaraldehyde and 2% paraformaldehyde. Purified kestose (GF2) was added twice with ethanol (twice 20 min each at 50, 60, 70, 80, 90, and 100%) and propylene oxide after being repeatedly washed with 0.1 M sodium carcodylate buffer. After dehydration, the concentration was gradually increased at 60 °C for two days, and the polymerization reaction was initiated in eponate812. After cutting the sample using an ultramicrotome (Ultracut UCT, Leica) and staining with uranyl acetate and lead citrate, the field emission SEM (JEM100F, JEOL, Japan) of the Basic Science Research Center of Kangwon National University [13].
**Trial registration**:	*Not applicable*
**Ethics**:	*Not applicable*
**Value of the Protocol**:	1. The pre-treatment helps in the extraction of high-purity fructooligosaccharide. 2. The alkaline combined enzymatic extraction protocol was efficiently applied to extract fructooligosaccharides from different agricultural bi-products with a higher yield ratio. 3. The Acetylated Extract-Extracted Fructooligosaccharide was liquefied in a mixed two-phase solvent system (LP: lower phase/PA: peak area of upper, 1:1, v/v).

## Description of protocol

The main focus of the new protocol is on the alkaline and enzymatic combined extraction-procedure for water-soluble oligosaccharide (kestose) from banana peel, quantification of hydrolyzed polysaccharide using high-performance liquid chromatography (HPLC-RID), further structural characterization of fructoligosacharide (FOS) using Fourier Transform Infrared (FTIR) spectroscopy, Nuclear Magnetic Resonance ((SEM).

## Extraction and manufacture of oligosaccharides from banana peel

**Fig. 1:**
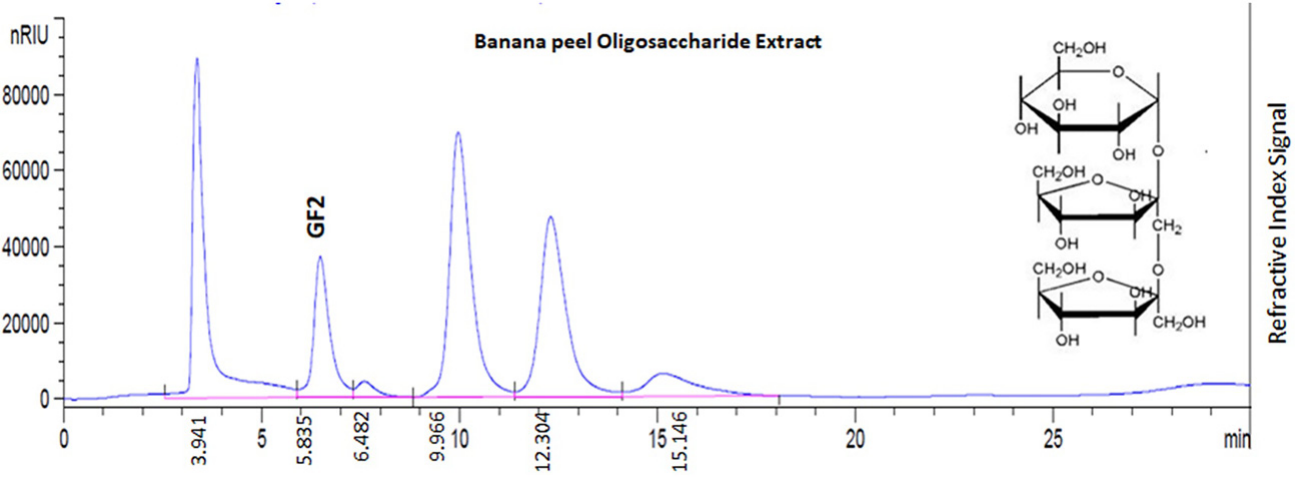
High-performance liquid chromatography with Refractive Index Detector (HPLC-RID) chromatogram of a standard fructooligosaccharide (FOS) from chicory root (F8052-Sigma-Aldrich, South Korea) (GF2 identified peek) compared with banana peel oligosaccharide extract (BPOE) [Determination conditions: mobile phase of acetonitrile and water (80:30, v/v), flow rate 1.0 mL/min, column temperature and RID temperature at 30 °C].

There is an increasing interest in using agricultural byproducts to find renewable and economically functional raw material sources in various application fields. Agricultural byproducts have nutrients that can be utilized to grow microorganisms that can create valuable enzymes, complex polysaccharides, and crude proteins. The following are examples of agricultural byproducts high in sucrose and can be utilized to make FOS. A similar argument might be made for peeled fruit such as mango, orange, pineapple, or some baguettes, as well as sugarcane, agave, corn, coconut, cassava, and any discarded leaves [14]. In this study, banana peel was used. After drying in an oven at 300 g of fresh banana peel, 50 g of banana powder was obtained using a grinder. Then, after degreasing by the Soxhlet method, a sample was obtained, treated with α-amylase and glucoamylase, centrifuged, and an enzyme-treated banana peel sample was obtained. Then, 99% ethanol was added, the precipitate was formed, lyophilized, and finally, about 20 g of a sample was obtained (Fig. 1).

## Isolation and identification of FOS (kestose) from BPOE

### Quantification of kestose (GF2) from BPOE using high-performance liquid chromatography with refractive index detector (HPLC-RID)

**Fig. 2:**
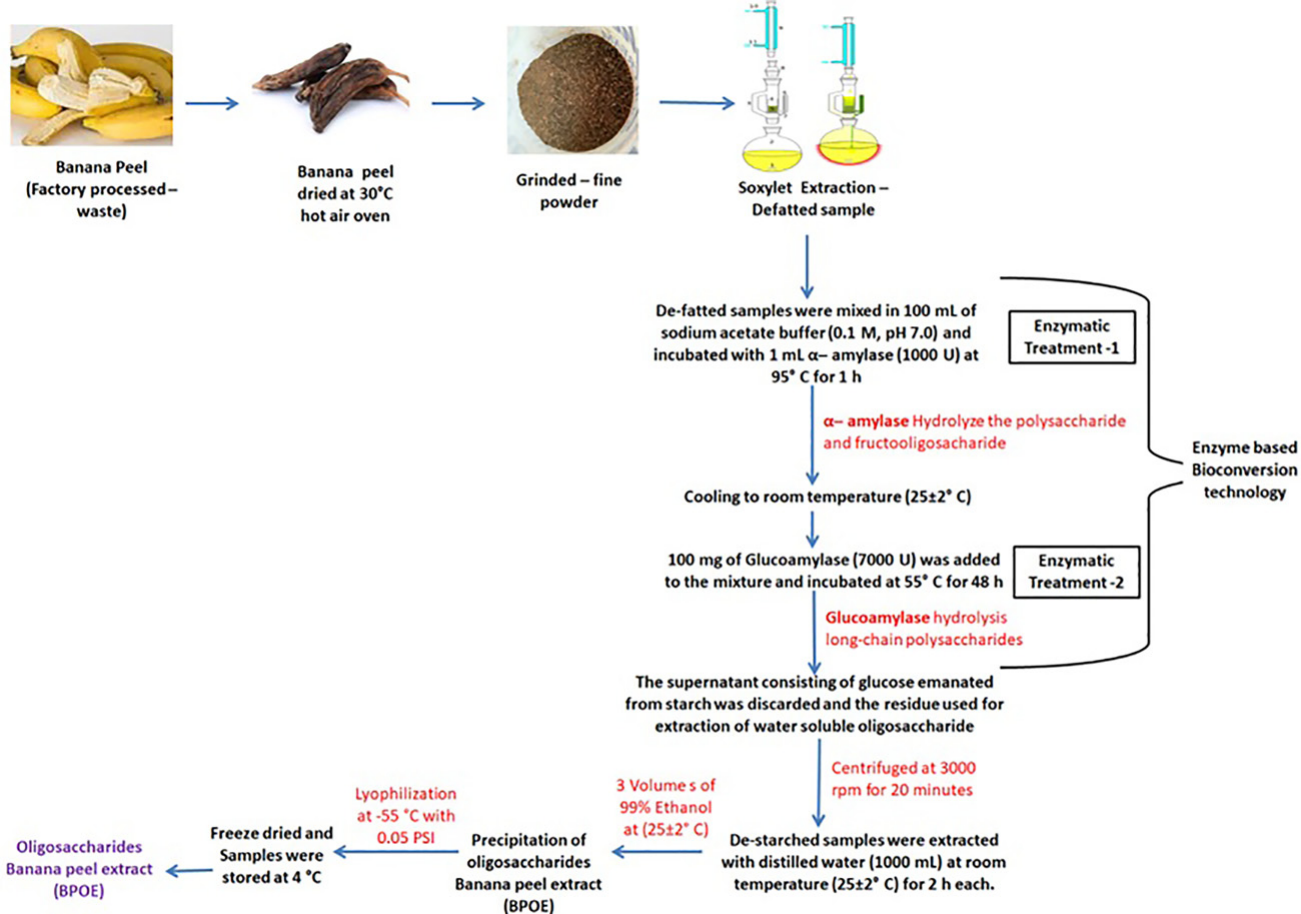
Process of total oligosaccharide extraction from banana peel.

In this study, the oligosaccharides were extracted from the peel, a byproduct of bananas, and used as a prebiotic material, kestose (GF2) has good probiotic efficacy among various types of fructooligosaccharides, was used as a marker. It was analyzed using HPLC-RID from the oligosaccharide extract (BPOE) isolated from the banana peel extract. As in Fig. 2, it was confirmed that BPOE was separated into a total of 5 peaks in the analytical chromatogram. When compared with the RT value (retention time) of the standard GF2, it was confirmed that the peak separated at RT 5.835 min was GF2 contained in BPOE. RT values ​​of the remaining four peaks were 6.482, 9.966, 12.304, and 15.146 min, respectively, and there were unidentified oligosaccharides. Similar studies reported using HPLC, GF2 was identified at the exact location in the yacon extract, and GF3 and GF4 were detected. Therefore, this study focused on GF2, known for its activity as a banana peel oligosaccharide extract prebiotic material, but the remaining GF3 and GF4 were not performed.

## Separation and purification of fructooligosaccharide – (kestose GF2) from the banana peel oligosaccharide extract (BPOE)

### Pre-column derivatization

#### Strategy for separation and purification of FOS

**Fig. 3:**
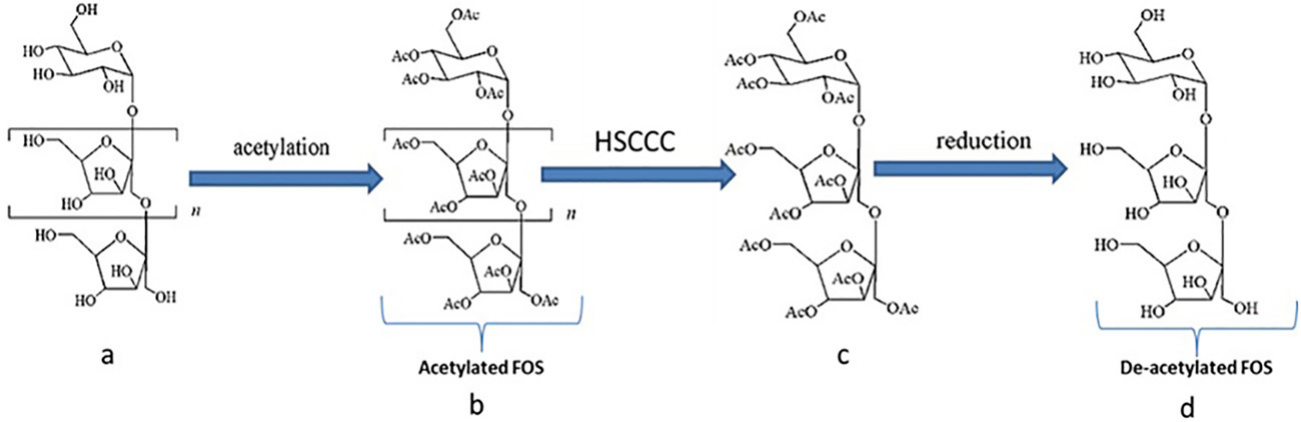
The acetylated kestose (GF2) was chromatographed using the HSCCC method. The following solvent systems were used: petroleum ether–n-butanol–methanol-water (3:2:1:4, v/v); stationary phase was in the upper phase; mobile phase was in the lower phase; flow rate was 2.0 mL/min; revolution speed was 800 rpm; retention of the stationary phase was 53.0 percent; sample load was 1 g, and ELSD did detection.

Traditional approaches are challenging to identify and separate FOSs due to their structural complexity, high polarity, and difficulty of detection. Furthermore, HSCCC separation is difficult because of their high hydrophilicity, which causes the target compounds to remain in the lower phase, even in n-butanol–water solvent systems. To comply with the HSCCC separation standards, a novel method for FOS preparative separation was used, in which the polarities of FOSs were lowered using acetylation. The compounds were then deacetylated, and FOS separation was confirmed (Fig. 3).

#### Selection of pre-column derivatization method

The suitable pre-column derivatization method was applied, resulting in ether-forming groups, which can be removed easily after the separation process. According to previous reports [15], the standard derivatization methods for hydroxyl groups are methyl, acetyl, and phenyl. In our research, the HSCCC was operated after the pre-column derivatization of FOS. Acetylation of hydroxyl groups in FOSs was chosen after weighing the experiment's difficulty, the amount of time it took to complete, and the extent of hydroxyl group substitutions.

### High-speed counter-current chromatography (HSCCC) separation and other conditions for separating kestose (GF2)

The selection of an appropriate two-phase solvent solution, which attained a basis for the efficient separation of the target compounds using HSCCC, requires an ideal range of KD values for the employed material, according to the KD value applied in a 0.5 to 2 range based on two-phase solvent. Accordingly, in this study, GF2 was isolated from the banana peel oligosaccharide extract using HSCCC, and a substance deduced as GF2, was collected at an elution time of 5.748. In this work, the FOS (Kestose) structures underwent acetylation. Based on knowledge gained from low-polar compound separation, a variety of solvent systems comprised of petroleum ether, n-butanol, methanol, and water were created with varying volume ratios (3:2:2:3, 3:2:1:4, and v/v) to generate the best KD for the target compounds. The hydroxyls became acetyls after acetylation, and their polarity was significantly lowered. The KD values were evaluated for the GF_2_ combination (Fig. 3).

### Determination of partition coefficient - distribution constant (KD)

The KD is less than 0.5; the solutes were eluted in close proximity to one another and the solvent front. The KD values of the two-phase solvent systems, including ethyl petroleum ether–n-butanol–methanol-water (3:2:2:3, v/v), were less than 0.5, resulting in poor peak resolution. The solutes eluted close to and near the solvent front in the two-phase solvent systems utilized for separation. The target compounds were isolated using two-phase solvent systems, but the separation time was too long. The two-phase solvent systems constituted of petroleum ether–n-butanol–methanol-water (3:2:1:4, v/v), with KD values between 0.5 and 2, suitable for separating the target chemicals. As a result, the HSCCC separation was done using the two solvent systems mentioned above. When two-phase solvent systems of petroleum ether–n-butanol–methanol-water (3:2:1:4, v/v).

### High-performance liquid chromatography coupled with evaporative light scattering detector HPLC-ELSD analysis to confirm the purified kestose (GF2) form BPOE

HPLC-ELSD analyzed the collected fractions of HSCCC. HPLC was applied to analyze the structure of the target compounds; FOS HPLC optimization. The design of the target compound was analyzed using HPLC, and the ELSD detector was chosen because of its excellent sensitivity and quick application time [16]. Samples eluted with solvent in the C18 chromatographic column have low resolution due to the strong polarity of FOS. The Xamide 100A column was chosen over the Inertsil NH2 Capillary Column for its high resolution and quick processing time. Several elution techniques, including isocratic acetonitrile-water and methanol-water elution, were investigated). The target compounds achieved satisfactory separation when the mobile phase comprised acetonitrile: water (80:20, v/v). The evaporating temperature was 80 °C, the Neb temperature was 90 °C, and the gas flow rate was 1.2 SLM. The column temperature was 30 °C; the mobile phase flow rate was 1.0 mL/min, the evaporating temperature was 80 °C, and the Neb temperature was 90 °C. As a result, a total of 24.9 mg of 1-kestose (GF2) compound was obtained from 1 g of sample, and the final fructooligosaccharide was confirmed to be kestose (GF2) as a result of HPLC-ELSD analysis (Fig. 3).

## Structural analysis and characterization of purified kestose (GF2)

### ^13^C-NMR

**Fig. 4:**
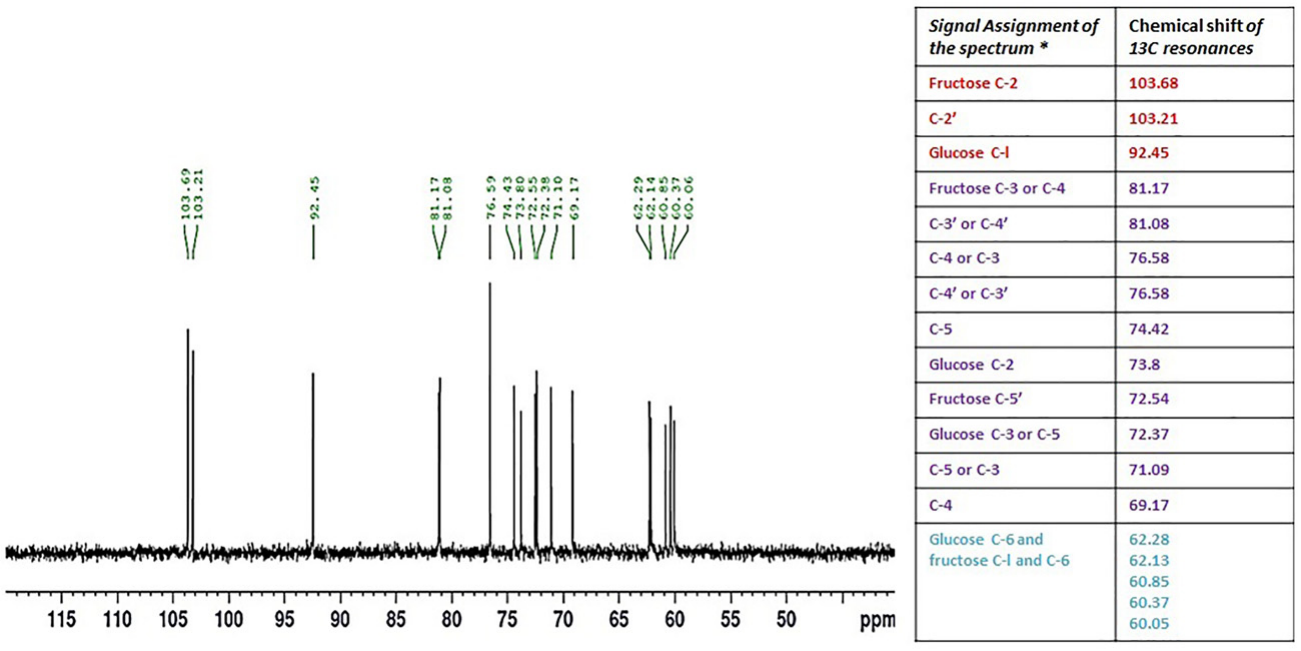
13C NMR spectra and chemical shifts of 1-kestose. Downfield of the 13C resonance. The general anomeric-carbon and primary CH2OH carbon assignments. These are tentative; the assignment of primes to numbers intended to differentiate between resonances of nuclei occupying similar positions specific identification. [Chemical shifts are expressed in ppm. ‘Measured in Deuterium oxide. ‘Data compared from WW. Binkley et al., 1972, H. C. Jarrel et al., 1979 and from the database (Biological magnetic resonance data bank (bmse001112- 1-kestose (C18H32O16)) - https://bmrb.io/metabolomics/mol_summary/show_data.php?id=bmse001112&whichTab=1)]. The table represent the 13C NMR Chemical shifts are expressed in ppm. downfield of the 13C resonance. The general anomeric-carbon and primary CH2OH carbon assignments. these are tentative; the assignment of primes to numbers is intended mainly to differentiate between resonances of nuclei occupying similar positions specific identification. ‘Measured in Deuterium oxide . ‘Data compared from Refs. [1,2] and from data base (Biological magnetic resonance data bank).

The ^13^C NMR spectrum of *HSCCC fraction* was shown in Fig. 4. The spectra of 1-kestose are shown in the figure. These molecules were used as reference materials, all of which contain *ᵟ*(2→ 1) linkages and are considered the smallest possible fructans and the structural building blocks of larger inulins (oligo-fructans). The 1-kestose carbon signals were assigned compared to reference materials and the previously reported chemical shifts of other 1-kestose [17].

The fructan spectrum exhibits the same broad signal at 62.28 that was regarded to be strong support for a (2 6) connection of D-Fruf moieties in pure 1-kestose from banana peel oligosaccharide extract [18]. Resonance for C-2 of -D-Fruf of 1-kestose has been set at 103.68 ppm, and an upfield shift of -0.1 ppm has been associated with the elongation of this kind of inulin [18]. The signals are absent in the regions from 105.0, which have been associated with C-2 of a 2,6-di-Osubstituted Fruf unit. The lack of signals at ä 102.3 and 98.9 for C-2 indicates the absence of â-D-Fruf as a reducing moiety.

The 13C NMR spectrum of sucrose in Fig. 4 shows an anomeric region with a signal at 92.45 ppm due to C-1 of an R-D-Glcp moiety. This signal is weaker than the anomeric C-2 of â-D-Fruf (ᵟ 103.21) in a relative ratio of 1:1. This ratio is observed in all reference materials. It changes pretty drastically once an overlapping of many ketoanomeric signals occurs. The region from ᵟ 60.05 to 62.28 comprises signals due to C-1 and C-6 of â-D-Fruf residues that sometimes are overlapped. The amount of a-D-Fruf in 1-kestose was found to be 60.85 ppm, which helps explain the C-1 and C6 signals in the pure 1-kestose from banana peel oligosaccharide extract spectrum (60.05). Resonance at 72.54 has also been linked to a Fruf residue at the end.

The absence of an anomeric signal of Fruf linked to internal glucose, the ᵟ 72.54 resonance in the purified extract might be attributed to an internal β2 → 1) Fruf residue. The resonance downfield at ᵟ 81.17 is assigned to a β(2→6) linkage or branched â-D Fruf residues; thus, ᵟ 81.17 in the purified extract spectrum could be due to either β-D-Fruf moiety. Another significant region for fructan structural elucidation is located from ᵟ 69.80 to 81.17, where C-5 signals are found. The HSCCC fraction presents two strong signals, one at ᵟ 81.1727 and the other at 81.0821 ppm. According to Lopez et al. [19] the former could be attributed to β(2→ 1) linkages and the latter to a β(2→ 6) linkage. The C-5 of the -D-Fruf residues linked through O-6 and the residue without a substituent at O-6 could cause these resonances [18]. A prior assignment by Sims et al. [18] demonstrating a structural resemblance of 1-kestose is consistent with the difference of 0.9 ppm between them. This shows that the structure of the pure banana peel extract might be comparable.

### ^1H^NMR

**Fig. 5:**
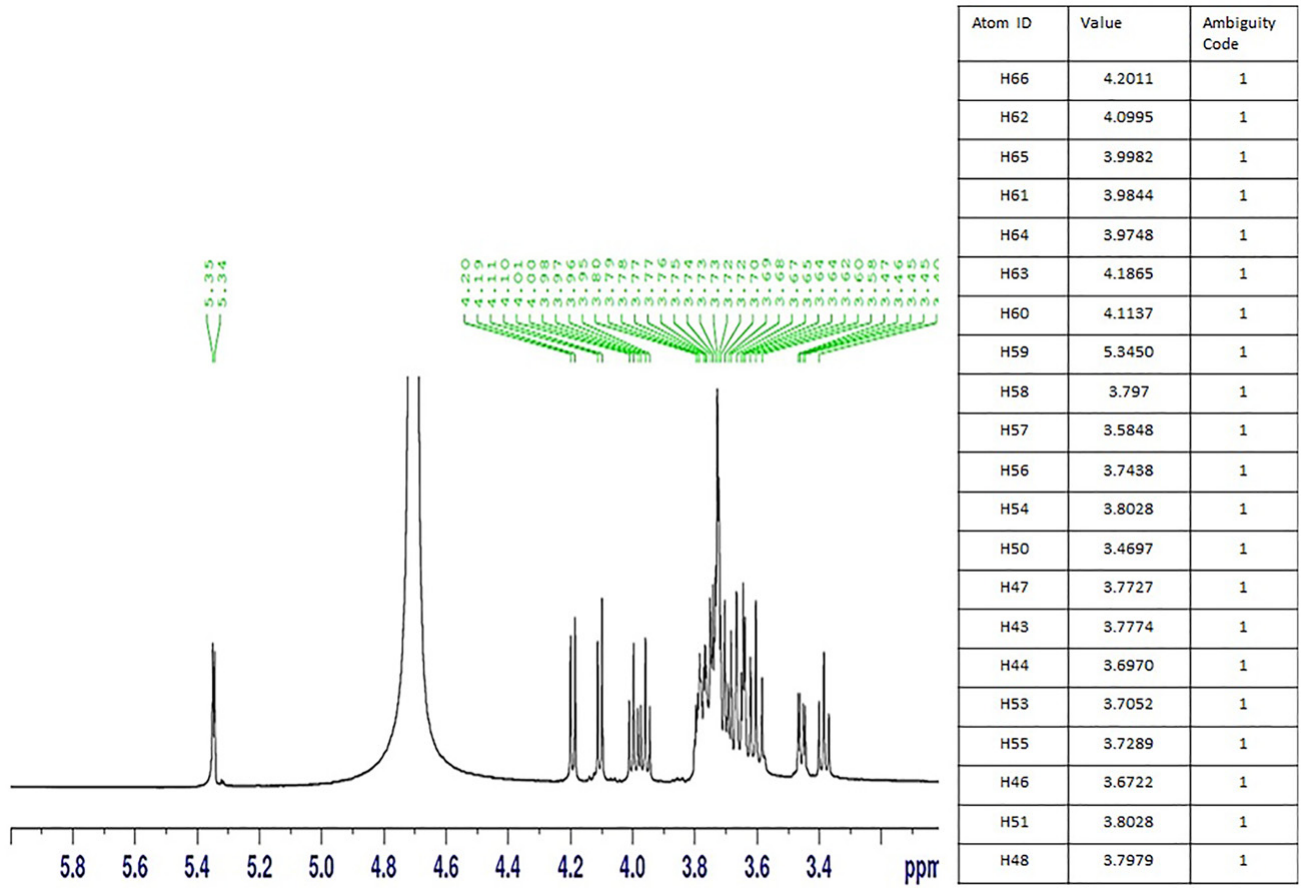
Proton NMR spectra and chemical shift of purified 1-kestose from bannan peel oligosaccharide extract using HSCCC. “Chemical shifts are expressed in ppm. Downfield of the proton resonance. ‘Measured in Deuterium oxide. ‘Data compared with (Binkley et al., 1969), and from database 1-kestose (C18H32O16) (Biological magnetic resonance data bank, https://bmrb.io/metabolomics/mol_summary/show_data.php?id=bmse001112). The table represent the h NMR (Proton) Chemical shifts are expressed in ppm. downfield of the proton resonance. ‘Measured in Deuterium oxide . ‘Data compared from Ref. [1], and from data base (Biological magnetic resonance data bank).

The ^1^H NMR spectrum of *HSCCC fraction* is shown in Fig. 5. The spectra of 1-kestose are shown in the same figure. The unambiguous assignment of H-l was a significant aspect in the entire 1H chemical shift assignment of 1. In the 1 D spectrum, its signal was the sole immediately distinguishable peak. H-2, H-3, H-4, and H-5 are the ‘H signals. Because both H-l and H-3 are connected to H-2, the spectrum of 1, cross-peaks between them was seen. The previous designations were supported by the discovery of cross-peaks between the H-3 and H-5 atoms of the two D-fructosyl units and between the H-4 and H-6 atoms of the two D-fructosyl units.

Proton NMR of fructans has been less studied than ^13^C NMR, resulting in very few reports on oligofructose being available [20]*.* Fig. 5 represents the ^1^H NMR spectrum of purified bannan peel extract. The chemical shifts for the H-1 of the R-D-Glcp residue can be found in the reference compounds of the kestose type at a value of -5.34. (55; 58; 59). According to these data, the resonance of H-1 fructan seems to agree very well with the chemical shift of internal glucose. The rest of the proton signals have been reported to appear in a narrow region between *ᵟ* 3.37 and 4.201. Proton assignments are very complex; however, their integration provides relevant information on the length of a polymer. Based on the proton integration of fructan (Fig. 5.), it can be established that *the purified 1-kestose (fructooligosaccharides)* from the banana peel (HSCCC fraction) is constituted of at least 16 residues; therefore, the glucose/fructose ratio is 1-15 at least.

### Two-dimension (2D)-homonuclear (H-H) J-resolved - correlation spectroscopy (COSY)

**Fig. 6:**
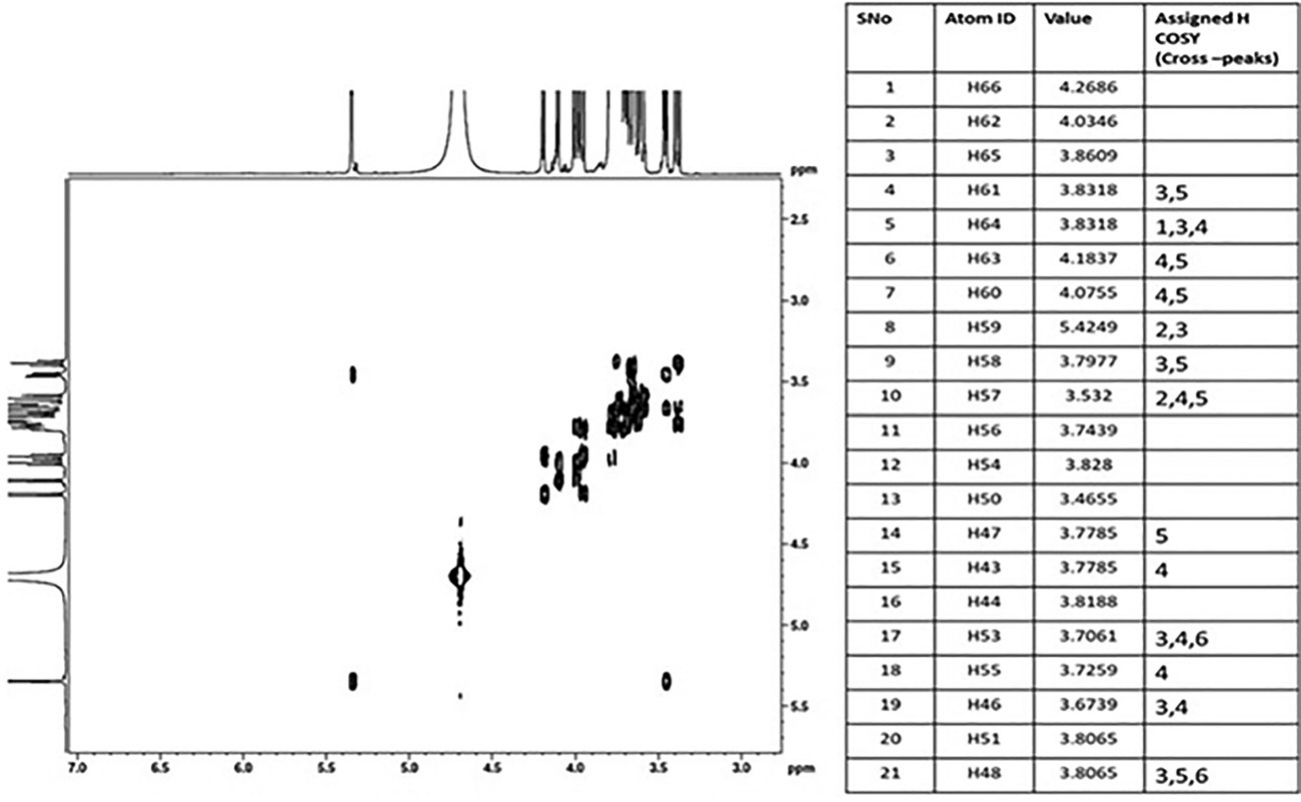
The Chemical-shift assignments are demonstrated using the Two dimensional (2D)-Homonuclear (H-H) J-resolved correlation spectroscopy (COSY) (2D NMR H-H) shift correlation and J-resolved spectra of 1. The unambiguous assignment of H-l was a critical aspect in the comprehensive ‘H chemical-shift assignment of 1 In the 1 D spectrum, its signal with sole discernible peak. 6B. Standard(NMR. Data for 1-kestose (C18H32O16) standard data retrieved from the biological magnetic resonance data bank) https://bmrb.io/metabolomics/mol_summary/show_data.php?id=bmse001112&whichTab=1.

The Overlapping or unresolved peaks made determining the multiplicities of the ‘H signals difficult, but the problem was handled by using the homonuclear, J-resolved experiment [21]. The investigation disperses the coupling pattern in the F 1 dimension, allowing exact chemical shifts and signal multiplicity to be determined even for overlapped signals. For example, the H-5 signals of all the sugar residues in 1 were absolutely undetectable in the 1D ‘H spectrum but were determined from the J-resolved spectrum (Fig. 6). The coupling patterns made it easier to assign signals that were too close to be distinguished from correlation spectra. Multiple cross-peaks for the H-5 signals have been observed in some correlation tests. Some extra weak peaks in this J-resolved spectrum could indicate the presence of two conformers in the Deuterium Oxide (D_2_O) solution. Multiple signals should, however, be avoided due to the predicted exchange rate.

## Morphological characterization of purified kestose (GF2)

### Particle size analysis (PSA)

The results based on particle size analysis of purified kestose (GF2) are shown in Sup. Fig. 3. the surface area of the particle sizes value was 6.36 μm, and these results showed similar results through TEM analysis. Changes in the morphology and particle size of purified kestose (GF2) may occur due to structural changes due to residues and related substances, which may also affect the functionality of other food uses [22]. In addition, the structural similarity of the purified kestose (GF2) based NMR analysis (Fig. 6), but minor variation was observed in the morphology of purified GF2. In particle size analysis (the state of a sample in the emulsion or powder state of a specific volume) reflects the change in the surface area exposed to moisture, hydration, and viscosity of the polysaccharide are the main factors. There is an inverse relationship between dissolution rate and particle size.

### Scanning electron microscopy analysis (SEM)

The purified kestose (GF2) displayed more hygroscopicity and aggregation than standard kestose, according to the microstructure of chicory-derived standard GF2 and purified kestose (GF2). On the other hand, the chicory-derived standard product GF2 has an irregular shape. Still, it is generally spherical, and purified kestose (GF2) has a round shape similar to that of the standard product kestose (GF2) but is highly aggregated on a rough surface. In the case of dried chicory-derived GF2, there were few aggregates, and it was confirmed that it was formed by individual spherical particles, which indicates the adsorption of minor moisture. In powder-dried chicory-derived standard kestose (GF2), the available size is between 29.84–58.54 µm, but there are some larger sizes of 108.56–124.88 µm. On the other hand, the general size of purified kestose (GF2) was 55.34–96.11 µm, which was generally more significant than that of standard kestose (GF2). Toneli et al. [23] evaluated the effect of humidity on the microstructure of spray-dried chicory-derived inulin and observed individual spherical particles. As the humidity increases, the particles begin to agglomerate. The powder becomes a continuous mass when exposed to an ambient environment with an aw (water activity) greater than 0.52. It can no longer be distinguished as individual particles [23].

## Declaration of Competing Interest

The authors declare that they have no known competing financial interests or personal relationships that could have appeared to influence the work reported in this paper.

## Data Availability

Data will be made available on request.
